# Practical Books for Hospital Workers

**Published:** 1894-04-21

**Authors:** 


### PRACTICAL BOOKS FOR HOSPITAL WORKERS.

Burdett's Hospital and Charities Annual, 1894. Being the Year-Book of Philanthropy. Fifth year. Edited by Henry C. Burdett, author of "Hospitals and Asylums of the World," &c., &c. (London: The Scientific Press, Limited, 428, Strand, W.C. New York: C. Scribner and Sons. Price, 5s.)

The "Hospital and Charities Annual" has established its position as the record of philanthropic work, and every year sees an addition to its size and completeness. The special articles in the fore part of the book are, as usual, of great interest. The narrative of the chief events which have occurred in the hospital world during 1893 is as readable as could be desired, and gives a clear and concise account of the work accomplished and the work that remains to do. We regret, however, that greater space is not devoted to discussing the need for paying wards in ordinary hospitals, which is becoming one of the most prominent points to be dealt with in both hospital supply and hospital finance. We should also like to see a discussion, if possible, by various experienced hands, on the different tests that should be used to prevent unsuitable persons obtaining medical relief at hospitals. In face of the constant deficit in the hospital purse, this is a question which should be dealt with vigorously and soon. But while noting these probably inevitable omissions, we have nothing but praise for what the "Annual" contains. The proportion of total expenditure which management should bear to maintenance is dealt with in a reasonable and practical spirit, and the recommendations err neither by excess nor by default. The tables of comparison of cost in the various hospitals are, as in past years, carefully done, and afford food for consideration, all the more that in this issue special care has been exercised to estimate as exactly as may be the average cost of the out-patients at each hospital. An interesting chapter gives the cost per patient, income, and expenditure, in the hospitals of the United States, a subject of which Mr. Burdett certainly knows more than any other man in this country. A closer knowledge of each other's methods will probably lead to exchange of improvements in both Britain and America, and we hope, therefore, that this chapter will attain an increasing importance in each issue of the "Annual." The development—as yet too slow—of trained nursing in workhouses forms another interesting record, which it is to be hoped will occupy a larger space in future issues. The list of Indian and Colonial hospitals increases in size and completeness every year, and shows in a gratifying way to what an extent intelligent medical charity is carried on throughout the British Empire. The "Hospital Annual" for 1894 is, in short, as full of interest as any of its predecessors, and even more full of information.

